# Diagnostic accuracy of cystoscopy and ultrasonography in the prenatal diagnosis of abnormally invasive placenta

**DOI:** 10.1097/MD.0000000000010438

**Published:** 2018-04-13

**Authors:** Yan Liu, Dazhi Fan, Yao Fu, Shuzhen Wu, Wen Wang, Shaoxin Ye, Rui Wang, Meng Zeng, Wen Ai, Xiaoling Guo, Zhengping Liu

**Affiliations:** a Department of Obstetrics; b Foshan Institute of Fetal Medicine, Southern Medical University Affiliated Maternal & Child Health Hospital of Foshan, Foshan, Guangdong; c Department of Epidemiology and Biostatistics, School of Public Health, Anhui Medical University, Hefei, Anhui, China.

**Keywords:** abnormally invasive placenta, cystoscopy, prenatal diagnosis, ultrasonography

## Abstract

The aim of this study was to compare the accuracy of cystoscopy and ultrasonography for the prenatal diagnosis of abnormally invasive placenta (AIP), including its subgroups: placenta accreta (PA), placenta increta (PI), and placenta percreta (PP).

A retrospective observational study including a total of 85 pregnant women at high risk for AIP underwent prenatal cystoscopy and ultrasonography evaluations. The sensitivity (Se), specificity (Sp), positive predictive value, negative predictive value, and exact diagnosed were calculated and compared for both cystoscopy and ultrasonography. Se and Sp values of cystoscopy and ultrasonography were compared by means of the McNemar test.

Of the 85 patients, there were 24 (28.2%) PA, 35 (41.2%) PI, 4 (4.7%) PP, and 22 (25.9%) nonadherent placenta. The mean maternal age and gestational age of delivery were 31.88 ± 4.42 years and 36.14 ± 1.84 weeks, respectively. No one was found to develop any complications with cystoscopy like urinary tract infection, or ureteral injury or perforations. Se in the diagnosis of AIP was 50.8% with ultrasonography and 61.9% for cystoscopy. Sp was 86.4% with cystoscopy and 72.7% for ultrasonography. In subgroups, Se with cystoscopy was 25.0%, 62.9%, and 100.0% in PA, PI, and PP, respectively, and 37.5%, 74.3%, and 100.0%, respectively, for ultrasonography; Sp remained unchanged with 86.4% for cystoscopy and 72.7% for ultrasonography. After McNemar test, no difference was found in either Se or Sp between cystoscopy and ultrasonography in AIP and its subgroups.

According to the depth of invasion, the diagnostic value of cystoscopy and ultrasonography is all conspicuous increased and they have similar test validity for prenatal diagnosis of AIP and its subgroups.

## 1 Introduction

Abnormally invasive placenta (AIP) is defined as trophoblastic attachment to the myometrium without intervening the decidua.^[1]^ According to the depth of invasion, it refers to the entire spectrum of conditions including placenta accreta (PA), placenta increta (PI), and placenta percreta (PP).^[2]^ It is associated strongly with the combination of prior caesarean section and placenta previa.^[3,4]^ Our recent study indicate that AIP is approximately 0.22% among deliveries in mainland China.^[5]^ Meanwhile, there is evidence that the occurrence of AIP has been steadily rising in the past several decades.^[6,7]^ It is regarded as one of the numerous adverse maternal and fetal–neonatal complications. The primary of maternal complication is life-threatening peripartum hemorrhage which can lead to hysterectomy, disseminated intravascular coagulation, multisystem organ failure, acute respiratory distress syndrome, and even death.^[8–10]^

Prenatal diagnosis of AIP can reduce the risk of maternal complications, such as, intraoperative blood loss and transfusion, the surrounding organs damage, cystotomy, hysterectomy, and other postoperative complications.^[11]^ Although commonly confirmed case at childbirth, antenatal diagnosis may be made with ultrasonography, magnetic resonance imaging (MRI), and cystoscopy.^[12,13]^ Ultrasonography is valuable tool in the prenatal diagnosis of AIP, whereas MRI is also said to be complementary to ultrasonography and may help in diagnosing placenta disorders.^[14]^ Although it is generally accepted that ultrasonography constitutes a highly reliable tool for diagnosing disorders of AIP, the performance is inconsistent in earlier published studies. Previous studies have reported that the sensitivities and specificities were ranging from 50% to 100% and 72% to 97% for the diagnosis of AIP, respectively, for ultrasound.^[15–17]^ Since 2003, the effectiveness of cystoscopy has been reported in some selected cases of PP with bladder wall or parametrium invasion.^[18–20]^ However, it has not found that the accuracy of cystoscopy in diagnosis of AIP in a larger sample size, especially in PA or PI.

Therefore, the aim of this study is to compare the sensitivity (Se), specificity (Sp), and accuracy of cystoscopy and ultrasonography for the prenatal diagnosis of AIP, including its subgroups PA, PI, and PP.

## 2 Material and methods

A retrospective analysis was performed of all patients referred for suspected AIP who had given birth at Southern Medical University Affiliated Maternal & Child Health Hospital of Foshan, Foshan, China from January 2012 to June 2016. This study protocol was endorsed by the Institutional Review Board of Southern Medical University Affiliated Maternal & Child Health Hospital of Foshan. Owing to the retrospective nature of the study, informed consent was not necessary, but personal data and confidentiality were prioritized. Our study included 85 patients who had been taken up by both prenatal ultrasonography and cystoscopy. Demographic and obstetric characteristics, such as maternal age, gestational age, gravidity, parity, previous abortions, and previous cesarean deliveries, were also collected in this study. Ultrasonography and cystoscopy were used by experienced obstetricians and urologists in AIP.

Ultrasonography was performed using the GE Voluson 730 (GE Medical Systems) with a 4.0 to 5.0 MHz or a 7.0 to 9.0 MHz transabdominal transducer for obese patients versus thin patients, respectively. When the ultrasonographic criteria were met (abnormalities of uterus-bladder interface, loss of the normal hypoechoic space, retroplacental placental thickness <1 mm, presence of placental lacunae),^[21,22]^ the diagnosis of AIP disorder was made.

**Figure 1:**
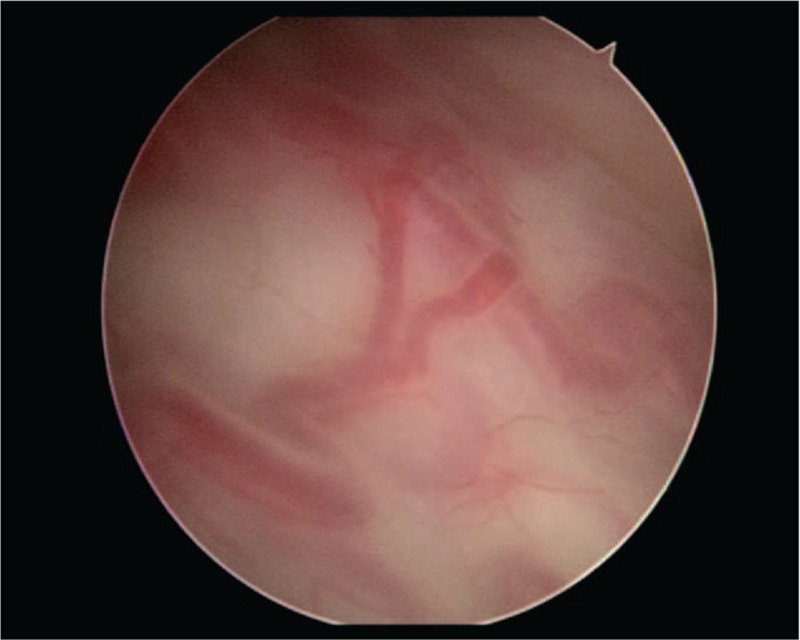
Cystoscopy in a case of abnormally invasive placenta. An augmented vascular network is present.

The procedure of cystoscopy (OLYMPUS CYF-2) was transurethral performed in the lithotomy position before induction of general anesthesia and commencement of the caesarean section by a urologist with >5 years of experience in the evaluation of placentation disorders and confirmed by another equally qualified urologist. The diagnosis of AIP disorder was made when cystoscopy showed placental vessels invading the urinary bladder mucosa.^[19]^Figure 1 showed an abnormal placentation by cystoscopy.

The criterion standard for diagnosis and differential of AIP was defined by clinical (difficult removal of the placenta and bleeding complications) basis and pathologic findings. During the delivery, if the placenta was easily removed without any bleeding complications, it was considered normal; if the uterine serosa or the adjacent organs had been reached by the placenta, it was considered PP; and if the placenta was found to be adherent to the myometrium and difficult to remove and bleeding ensued after attempting at placenta delivery,^[17]^ it was suspected PA or increta and the abnormal tissue was confirmation of the final histology.

### 2.1 Statistical analysis

Qualitative data were expressed in number and percentage; quantitative data were expressed in mean and standard deviation. Se, Sp, positive predictive value (PPV), negative predictive value (NPV), and exact diagnosed (ED) were calculated for both cystoscopy and ultrasonography. Se and Sp values of cystoscopy and ultrasonography were compared by means of the McNemar test. To compare the effectiveness of them in the depth of invasion, subgroups data (PA, PI, and PP) were recalculated respectively. Statistical analysis was performed using R statistical software (Version 3.2.5; www.r-project.org).

## 3 Results

### 3.1 Characteristic of the patients

Eighty-five patients suffered both cystoscopy and ultrasonography to explore suspected AIP. All of them chose an elective cesarean section. Maternal sociodemographic characteristics and clinical information were given in Table 1. The mean maternal age and gestational age of delivery were 31.88 ± 4.42 years and 36.14 ± 1.84 weeks, respectively. There were 24 (28.2%) PA, 35 (41.2%) PI, 4 (4.7%) PP, and 22 (25.9%) nonadherent placenta. In the 4 PP cases, 1 had a total hysterectomy and 3 had a subtotal hysterectomy to control the postpartum hemorrhage. No one was found to develop any complications with cystoscopy like urinary tract infection, or ureteral injury or perforations.

**Table 1:**
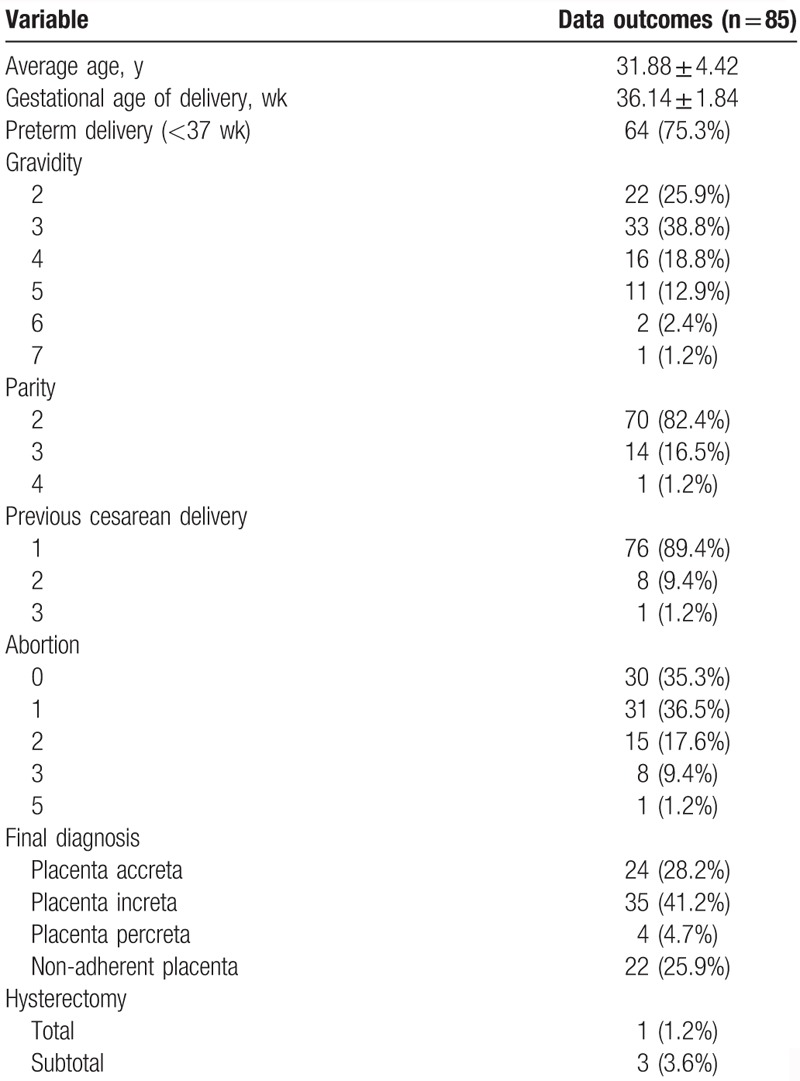
Summary of clinical information of included patients.

### 3.2 Consistency result

**Figure 2:**
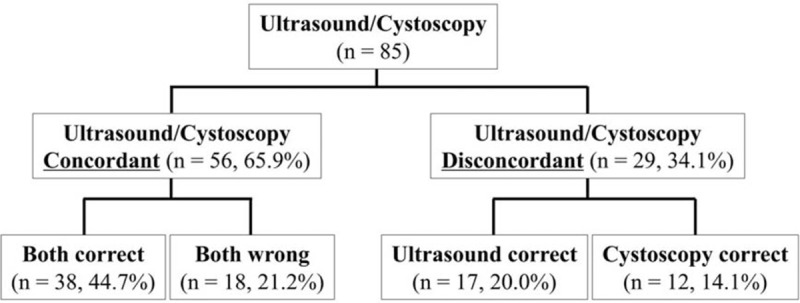
Concordance between cystoscopy and ultrasonography.

Ultrasonography and cystoscopy were concordant in 56 of 85 cases (65.9%). In 38 patients, both ultrasonography and cystoscopy were correctly diagnosed, and in 18 patients both were mistaken. There was a disagreement between ultrasonography and cystoscopy in 29 patients, and the ultrasonography diagnosis was correct in 17 of these patients. Sixteen false-negative results given by cystoscopy were correctly diagnosed by ultrasonography. On the contrary, in 12 of 29 patients cystoscopy correctly invalidated a diagnosis by ultrasonography (4 false-positive and 8 false-negative diagnosis). These results were illustrated in Figure 2.

### 3.3 Diagnostic accuracy

The Se, Sp, PPV, NPV, and ED associated with the use of ultrasonography and cystoscopy were listed in Table 2. It revealed diagnostic validity of ultrasonography in comparison to cystoscopy with Se, Sp, PPV, NPV, and ED of 61.9% (95% confidence interval [CI], 48.8–73.9); 72.7% (95% CI, 49.8–89.3); 86.7% (95% CI, 73.2–95.0); 40.0% (95% CI, 24.9–56.7); and 52.9% (95% CI, 41.8–63.9) for ultrasound, and 50.8% (95% CI, 37.9–63.6), 86.4% (95% CI, 65.1–97.1); 91.4% (95% CI, 76.9–98.2); 38.0% (95% CI, 24.7–52.8); and 60.0% (95% CI, 48.8–70.5) for cystoscopy, respectively.

**Table 2:**
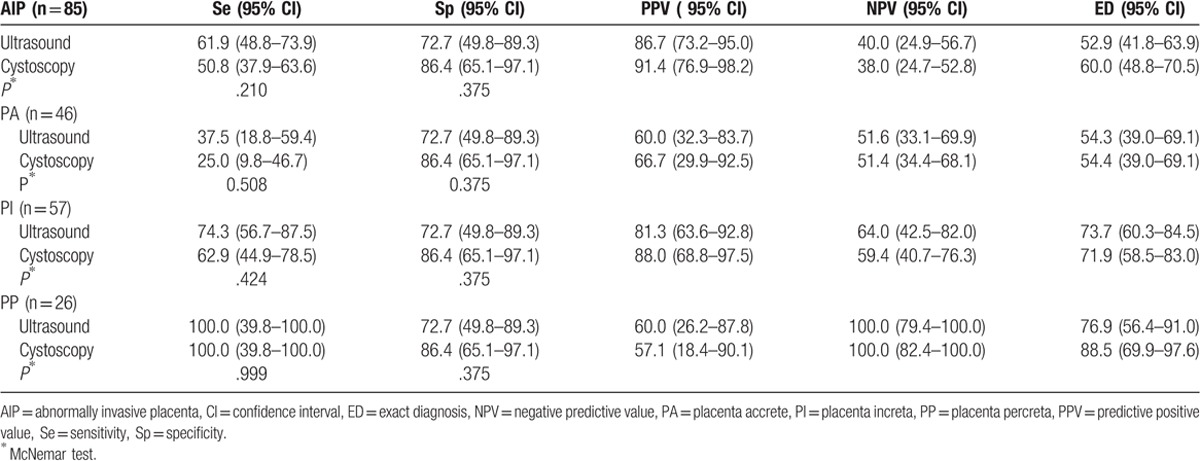
Sensitivity and specificity of ultrasonography and cystoscopy in abnormally invasion placenta and subgroups.

### 3.4 Subgroups analysis

In subgroups, the overall performance of ultrasonography for the antenatal detection of PA was as follows: Se, 37.5% (95% CI, 18.8–59.4); Sp, 72.7% (95% CI, 49.8–89.3); PPV, 60.0% (95% CI, 32.3–83.7); NPV, 51.6% (95% CI, 33.1–69.9); and ED, 54.3% (95% CI, 39.0–69.1). The results of cystoscopy were as follows: Se, 25.0% (95% CI, 9.8–46.7); Sp, 86.4% (95% CI, 65.1–97.1); PPV, 66.7% (95% CI, 29.9–92.5); NPV, 51.4% (95% CI, 34.4–68.1); and ED, 54.3% (95% CI, 39.0–69.1) (Table 2).

For PI, the overall Se, Sp, PPV, NPV, and ED of ultrasonography were 74.3% (95% CI, 56.7–87.5), 72.7% (95% CI, 49.8–89.3), 81.3% (95% CI, 63.6–92.8), 64.0% (95% CI, 42.5–82.0), and 73.7% (95% CI, 60.3–84.5) compared to 62.9% (95% CI, 44.9–78.5), 86.4% (95% CI, 65.1–97.1), 88.0% (95% CI, 68.8–97.5), 59.4% (95% CI, 40.7–76.3), and 71.9% (95% CI, 58.5–83.0) for cystoscopy, respectively (Table 2).

The Se, Sp, PPV, NPV, and ED in the detection of PP were 100.0% (95% CI, 39.8–100.0), 72.7% (95% CI, 49.8–89.3), 60.0% (95% CI, 26.2–87.8), 100.0% (95% CI, 79.4–100.0), and 76.9% (95% CI, 56.4–91.0) for ultrasound, and 100.0% (95% CI, 39.8–100.0), 86.4% (95% CI, 65.1–97.1), 57.1% (95% CI, 18.4–90.1), 100.0% (95% CI, 82.4–100.0), and 88.5% (95% CI, 69.9–97.6) for cystoscopy, respectively (Table 2).

**Figure 3:**
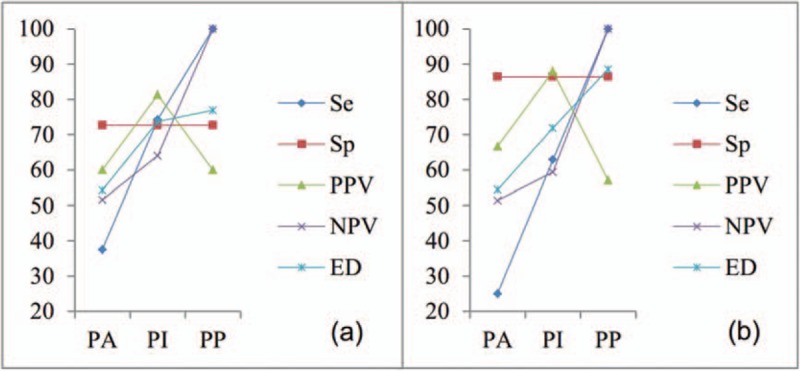
The efficacy of ultrasonography and cystoscopy in diagnosing of PA, PI, and PP. (A) ultrasonography; (B) cystoscopy. ED = exact diagnosis, NPV = negative predictive value, PA = placenta accrete, PI = placenta increta, PP = placenta percreta, PPV = predictive positive value, Se = sensitivity, Sp = specificity.

According to the depth of invasion (PA, PI, and PP), Se, NPV, and ED were all increased; Sp remained unchanged; and PPV increased at first and then decreased subsequently for ultrasonography and cystoscopy (Table 2, Figure 2). After McNemar test, there was no difference in either Se or Sp between ultrasonography and cystoscopy in AIP, including PA, PI, and PP (Fig. 3).

## 4 Discussion

This study was to compare the accuracy of the most widely available diagnostic tool (ultrasound) and cystoscopy for the prenatal diagnosis of AIP and its subgroups, including PA, PI, and PP. Ultrasonography and cystoscopy showed high Se, Sp and PPV, but the NPV was relatively low. According to the depth of invasion, the diagnostic value of ultrasonography and cystoscopy were all conspicuous increased. Our study also showed ultrasonography and cystoscopy appear to have similar test validity for prenatal diagnosis of AIP and its subgroups.

Although the precise staging of AIP was dependent on pathological report, reliable preoperative investigations would help obstetricians to differentiate the point of invasion placenta and normal tissue. Ultrasonography has been usually employed as the primary imaging modality for the antenatal diagnosis of AIP.^[23]^ In 1992, the first prospective ultrasonography diagnosis in patients with AIP was reported by Finberg and Williams.^[24]^ They performed it to use in 34 patients with placenta previa and a history of ≥1 cesarean sections. Eighteen were interpreted as positive, and 14 (77.8%) had tissue confirmation. Meanwhile, they made a negative diagnosis in 16 patients, of whom 1 (6.3%) had AIP at delivery.

Subsequently, several original researches and meta-analysis,^[16,25,26]^ including retrospective and prospective studies, were reported to assess the performance of ultrasonography in the prenatal diagnosis of AIP. Although most of these studies suggested that ultrasonography has a primary role in screening women at risk for AIP, there were still some inconsistent to be worth to explore. Previous studies showed that the results of sensitivities and specificities of ultrasonography in the prenatal diagnosis of AIP were great variability, from 13% to 100% and 35% to 100%,^[24,26,27]^ respectively.

In this study, Se and Sp were 61.9% and 72.7%, respectively. These findings were in agreement with the results of the previous studies.^[15–17]^ However, the result of Se was significant variability from 37.5% to 100.0% in different depth of invasion. This may be clarified why the effectiveness of ultrasonography for prenatal diagnosis of AIP was differences in the previous studies.

AIP accounts for 33% to 50% of all emergency peripartum hysterectomies,^[28,29]^ most of them attributed PP. Timely recognition of AIP likely reduced maternal morbidity. Furthermore, when the women were suffered by PP, total hysterectomy could be reduced substantially if careful peripartum planning is done.^[30,31]^ The surgical approach would be improved by knowing the exact location of the PP and the extent of invasion.

Through the previous lower uterine scars, PP usually invaded the posterior wall of bladder. Unlike the painless third trimester antepartum hemorrhage common with placenta previa, vaginal bleeding of PP was more likely to be painful due to invasion of the hemorrhaging placental tissue into the uterine wall.^[32]^ However, in only about 25% of women occurred gross hematuria when their bladder was invaded by placenta.^[33]^ Therefore, most pregnant women of PP that involved the bladder were recognized only at the time of delivery.

Cystoscopy has taken advantage of many physicians to determine bladder invasion and to evaluate the anatomical extension of the tumor.^[34–36]^ In PP, obstetricians and urologists have been reported the effectiveness of cystoscopy in several case reports.^[18–20]^ However, in a larger sample size, especially in PA or PI, the effectiveness of cystoscopy has not found to be reported. Our study revealed that the Se and Sp of AIP were 50.8% and 86.4%, respectively. These results were in conformity with the ultrasound. Despite the fact that there was no difference in the Sp, it was higher (86.4% vs. 72.7%) in cystoscopy than ultrasound. Meanwhile, the most reliable sign for determining directly the exact location of the PP and the extent of invasion was visible during cystoscopy before commencement of the cesarean section.

There were some limitations to our study. It was a retrospective and longitudinal study with an inherent bias, leaving us unable to control for significant variables. Another potential limitation of this study was that the data were collected about a 5-year time frames, and >1 obstetrician and pathologist were involved over the period examined and study reported high interobserver discrepancy, but this reflected the real world. Further prospective, multicenter, and well-defined studies might be needed to assess the real accuracy of cystoscopy in prenatal diagnosing of AIP.

In conclusion, depending upon the depth of invasion, the diagnostic value of cystoscopy and ultrasonography were all conspicuous increased and they appeared to have similar test validity for prenatal diagnosis of AIP and its subgroups.

## Acknowledgments

The authors thank Shilin Zhang, from the Department of Urology, for helping with his knowledge of cystoscopy.

## Author contributions

Z.L. and DF conceived the initial idea and developed and designed the project; Y.L. and D.F. wrote the first draft, and the other authors edited subsequent versions of the draft; Y.F., S.W., S.Y., and R.W. contributed to the patient data collection; D.F., W.W., and R.W. performed the data analysis; M.Z., W.A., and X.G. critically revised the manuscript.

**Conceptualization:** Yan Liu, Dazhi Fan, Meng Zeng, Xiaoling Guo, Zhengping Liu.

**Data curation:** Dazhi Fan, Yao Fu, Shuzhen Wu, Wen Wang, Shaoxin Ye, Rui Wang, Meng Zeng, Wen Ai.

**Formal analysis:** Dazhi Fan, Wen Wang.

**Project administration:** Zhengping Liu.

**Visualization:** Zhengping Liu.

**Writing – original draft:** Yan Liu, Dazhi Fan.

**Writing – review & editing:** Dazhi Fan, Yao Fu, Shuzhen Wu, Wen Wang, Shaoxin Ye, Rui Wang, Meng Zeng, Wen Ai, Xiaoling Guo, Zhengping Liu.
